# Pelvic Version; Report of Cases, with Remarks

**Published:** 1881-12

**Authors:** Chas. C. F. Gay


### Pelvic Version; Report of Cases, with Remarks.

By Chas. C. F. Gay, M. D.

### CASE I.—SHOULDER PRESENTATION; VERSION.

Mrs. H., æt. 28 years; American; multipara; third pregnancy; former labors natural; patient in charge of Dr. Gould, and in labor twelve hours. Membranes had ruptured with escape of water some time prior to my visit. Pains were feeble; chloroform was given, when I passed up my right hand and brought down one foot. By making considerable traction, in fifteen minutes the woman was delivered of a still-born female child. Mother recovered.

CASE II.—VERTEX PRESENTATION; VERSION.

Mrs. ———; Irish; multipara; sixth labor, all difficult and still-births; vertex presented; Drs. Diehl and Wetmore in attendance. Forceps had been used and craniotomy attempted; labor had lasted several hours. I passed up my left hand, seized a foot, but could not withdraw either the foot or my closed hand clasped around the child's leg, until my hand was opened; applied blunt hook above the child's heel, drew down the foot, effected version, and delivered the head by using great force. Child had been dead at least two or three hours. There were hour-glass contractions, and the conjugate diameter judged to be less than three inches. Chloroform employed. Recovery of the patient.

CASE III.—SHOULDER PRESENTATION; VERSION.

Mrs. ———; English; multipara; sixth labor; Dr. J. C. Green called me to this case; in labor several hours. Membrane had been ruptured spontaneously, and waters had escaped. Under chloroform I turned with considerable difficulty; much more indeed than would have been experienced had the membranes remained intact. Brought down one foot and delivered the woman of a still-born child. Mother had a good recovery.

CASE IV.—VERTEX PRESENTATION; PROLAPSE OF CORD; VERSION.

Mrs. M.; German; æt. 32 years; sixth labor; Dr. Diehl in attendance; woman in labor all day, and at first attended by a midwife. Pains had ceased at evening; large portion of cord was prolapsed. Placed patient in knee and elbow position, and reposited the cord, which would not so remain on account of absence of pains. Forceps applied without effecting delivery. Under chloroform, with my right hand one foot was reached and then the other. The body of the child still-born, delivered, and the case given to Dr. Diehl to complete. There was much hemorrhage; patient recovered.

CASE V.—PARTIAL VERTEX PRESENTATION, WITH RIGHT HAND PROTRUDING, A LITTLE IN ADVANCE OF HEAD; VERSION AFTER FAILURE WITH FORCEPS.

Mrs. ——— ; German , æt. about 32 years; fourth labor; in charge of Dr. Diehl; midwife had been in attendance several hours; waters had escaped; forceps applied, but strong traction during pain was of no avail. I passed up my right hand, brought down one foot, turned and delivered the woman of a dead male child. Mother recovered without a bad symptom.

CASE VI.—RIGHT HAND AND CORD PRESENTING; VERSION.

Patient æt. 40 years; in her sixth labor; in charge of Dr. Gould; had been in labor only three hours; membranes ruptured and cord prolapsed. Under an anæsthetic I introduced my right hand, brought down one foot and delivered this woman of a dead boy. Mother recovered without any untoward symptom.

CASE VII.—TRANSVERSE PRESENTATION; VERSION.

Mrs. A.; æt. 32 years; fifth labor; cross presentation; has been in labor about eighteen hours; pains have now ceased; patient in care of Dr. Folwell. Under chloroform I passed my right hand into the uterine cavity; seized one foot and brought it down; delivered both lower limbs, then turned the case over to the doctor for completion of labor. There was much hemorrhage; both mother and child lived.

CASE VIII.—TWINS; FIRST CHILD DELIVERED ALIVE NATURALLY; HAND PRESENTATION OF SECOND CHILD; VERSION.

Mrs. W.; æt. 26 years; primipara; in charge of Dr. Damback; had been in labor during the entire night; a living child had been delivered; pains had ceased and waters discharged. On examination I found a right hand presenting, and ascertained that the child was dead. Under chloroform I turned, by drawing first one foot down and then the other, but not without much difficulty and effort. The delivery of a double placenta soon followed. The patient made a good recovery.

### REMARKS.

These cases are arranged chrononogically. Death of the fœtus, in two of the cases, was ascertained before any attempt was made at version. In one case only was there a living child delivered. Five versions out of eight cases resulted in the death of the fœtus, which gives sixty-two per cent. of fatal cases as the result of pelvic version. It appears, therefore, that while pelvic version inflicts no harm upon the mother, it is quite destructive to the child, and leads one to cast about for some method which shall be equally safe for both mother and child.

It was Celsus who recommended extraction of the fœtus by the pelvic extremity, but only after the fœtus had ceased to live. This precept prevailed until the time of Ambrose Parē. He was the first to suggest pelvic version on the living fœtus (Chailly). I do not presume to offer any suggestion or new method as an improvement of the old, for the safe delivery of the fœtus in cases requiring version, but I wish in this connection to direct attention to a method recommended by Dr. E. R. Maxon*, an account of which was published in the year 1867, and which at the time it was made public, obtained the endorsement of the late Sir James Y. Simpson. Maxon's plan consists in a change as he says, from an "abnormal into a perfectly natural presentation, without risk to mother or child, by placing the mother on her knees for from three to five minutes, on pillows of folded quilts, with the head and shoulders low, as suggested by Thomas, to return a prolapsed cord."

If this plan be feasible, I trust it may have trial. Although my attention was directed to it some years since, I must confess that when called to the bedside of the parturient woman, that Maxon's method has escaped my memory, but now, since again reverting to it, I shall put the plan into execution and test its merits at the first opportunity.

---

* *Medical Record*, N. Y., p. 661, vol. x, 1875.

